# Iranian and American Moral Judgments for Everyday Dilemmas Are Mostly Similar

**DOI:** 10.3389/fpsyg.2021.640620

**Published:** 2021-03-30

**Authors:** Aryan Yazdanpanah, Sarvenaz Soltani, Fatemeh Sadat Mirfazeli, Seyed Vahid Shariat, Amin Jahanbakhshi, Faraneh GhaffariHosseini, Kaveh Alavi, Parisa Hosseinpour, Parisa Javadnia, Jordan Grafman

**Affiliations:** ^1^Cognitive Systems Laboratory, Control and Intelligent Processing Center of Excellence (CIPCE), School of Electrical and Computer Engineering, College of Engineering, University of Tehran, Tehran, Iran; ^2^Mental Health Research Center, School of Behavioral Sciences and Mental Health (Tehran Institute of Psychiatry), Iran University of Medical Sciences, Tehran, Iran; ^3^Department of Neurosurgery, Skull Base Research Center, Rasool-e-Akram Hospital, Iran University of Medical Sciences, Tehran, Iran; ^4^Faculty of Medicine, Rasool-e-Akram Hospital, Iran University of Medical Sciences, Tehran, Iran; ^5^Shirley Ryan AbilityLab, Departments of Physical Medicine and Rehabilitation, Neurology, Cognitive Neurology and Alzheimer's Center, Chicago, IL, United States; ^6^Department of Psychiatry, Feinberg School of Medicine and Department of Psychology, Weinberg College of Arts and Sciences, Northwestern University, Chicago, IL, United States

**Keywords:** moral judgments, moral cognition, moral vignettes, cross-cultural differences, everyday moral dilemmas

## Abstract

Moral judgment is a complex cognitive process that partly depends upon social and individual cultural values. There have been various efforts to categorize different aspects of moral judgment, but most studies depend upon rare dilemmas. We recruited 25 subjects from Tehran, Iran, to rate 150 everyday moral scenarios developed by Knutson et al. Using exploratory factor analysis (EFA), we observed that the same moral dimensions (except socialness dimension) were driven by the same moral cognitive factors (norm violation, intention, and social affect) in Iranian vs. American studies. However, there were minor differences in the factor loadings between the two cultures. Furthermore, based on the EFA results, we developed a short form of the questionnaire by removing eleven of the fifteen scenarios from each of the ten categories. These results could be used in further studies to better understand the similarities and differences in moral judgment in everyday interactions across different cultures.

## Introduction

Moral judgment is a conscious cognitive process that includes decision-making and ethical considerations in keeping with regional social and individual cultural values (Aybek et al., 2015). Moral actions often require putting other's benefits before self-desires (Crockett, 2013). Moral decision-making sometimes requires a person to arbitrarily decide how to act in an actual or in a simulated moral scenario—which is called a moral dilemma (Garrigan et al., 2018).

Scientific contributions to studying moral cognition and moral judgments involve biology, psychology, anthropology, and philosophy (Hofmann et al., 2014). More recently psychologists and cognitive neuroscientists have designed complex research often involving neuroimaging methods that supersede prior approaches that relied upon surveys. These studies led to an increased understanding of how ethical sensitivity and moral decision making is developed, varied among different cultures, which factors affect moral decisions, and how moral standards are related to social and individual interactions (Greene, 2015).

To complement behavioral studies examining how moral decisions are formed, functional neuroimaging and lesion mapping studies of patients have begun to reveal the neural processes that are involved in different aspects of moral judgments (Moll et al., 2005; Garrigan et al., 2016). There have been various efforts to categorize these different aspects of moral cognition (e.g., Greene et al., 2001; Mendez et al., 2005; Beauchamp et al., 2013). For example, Moll et al. (2005) suggested that moral judgment is based on selfish and altruistic motives, emotions, norms, etc. (Moll et al., 2005). Furthermore, Knutson et al. (2010) have reported that the three cognitive factors underlying moral judgment are intention, norm violation, and social affect.

Moral judgments are often made quickly after the first interaction with people based on instant impressions of another person's personal and social behaviors (Uhlmann et al., 2015). Some suggest that personal ethical insights are mostly affected by self-conscious emotions while decisions that are related to social issues are influenced by cognitive orientation (Greene et al., 2004).

Alongside cultural differences, in several studies, social features and individual demographic characteristics including age, sex, education level, ideologies, religious beliefs, and stage of moral cognitive development have been shown to have undeniable effects on moral decision-making (Glover et al., 1997).

Traditionally, most academic studies of moral judgment have employed complex and rare dilemmas (Greene et al., 2001; Greene and Haidt, 2002; Koenigs and Tranel, 2007; Graham et al., 2009, 2016). A well-known example is the Trolley Car Dilemma but except for Drone operators in combat situations, it would be very rare for the average person to be required to make such a decision. It suggests that for the typical experiment using such a dilemma, a research participant probably uses reasoning, social inference, and a range of untested ideas about the preferred response. It is rarer to encounter studies requiring people to make judgments about moral decisions emerging from everyday experience (Knutson et al., 2010). Given that these sorts of dilemmas are the one most participants would know the preferred way to react, there remains the need for additional research regarding everyday moral decision making. To that end, using moral dilemmas in different emotion-based and action categories seems a promising way to investigate everyday moral decision-making. For example, a moral scenario might be more selfish than social, more intentional than accidental, or more emotional.

Therefore, we aimed to validate and then abbreviate the American-based everyday moral vignettes prepared by Knutson et al. (2010) in Iran to see whether there are culturally specific factors influencing everyday moral judgment. These moral vignettes include 150 scenarios within different moral categories and measure different dimensions within each scenario of the categories.

## Methods

### Participants

Twenty-five healthy adults [mean age = 30.1 +/– 6.09 (SD); 15 females, 10 male; all had university-level educations, 16–22 years of education] were recruited from staffs of two hospitals (mostly health care professionals and other employees) in two different regions of Tehran with different socio-economic backgrounds. Participants did not report any history of major psychiatric or neurological disorders such as bipolar disorder, epilepsy, brain tumor, etc. Informed consent was obtained from participants.

### Measures

We used a paper-and-pencil version of the moral vignettes task that was developed by Escobedo (2009) and condensed and standardized by Knutson et al. (2010). The moral vignettes consist of 150 short scenarios in 10 main emotion-based and action categories including compassion, being guilty, honesty, being harmful to someone, lying, being regretful, behaving sneaky, being tempted, taking something from someone, and being unfaithful. For example, in the compassion category, an example of one vignette is: “About 10 years ago I was friends with a woman who was a musician. She was struggling and she wasn't making any money. So I decided to lend her some money.” Each vignette is rated on 13 dimensions (emotional intensity, emotional aversion, harm, self-benefit, other-benefit, pre-meditation, illegality, social norm violations, socialness, frequency, personal familiarity, general familiarity, and moral appropriateness) using a 7-point Likert scale.

### Procedure

This study consisted of 8 steps: 1. Translation and cultural adaptation of the moral vignettes, 2. Data collection 3. Exploratory factor analysis (EFA) to determine the underlying moral cognitive factors in Farsi, 4. Cross-cultural comparison of the underlying moral cognitive factors, 5. Reducing the number of vignettes in each category, 6. Comparing the reduced questionnaire's underlying moral cognitive factors between Iranian and American participants, 7. Exploratory factor analysis on the categories, and 8. Measurement of Cronbach's alpha.

**Step 1. Translation and cultural adaptation:** First, moral vignettes from Knutson et al.'s Behavioral norms for condensed moral vignettes (Knutson et al., 2010) were translated to Farsi with the help of an expert bilingual translator and it was checked if the scenarios were plausible in Iranian culture or could potentially take place within the cultural norms and rules of the country by an expert panel consisting of four psychiatrists. Only 4 scenarios out of a total of 150 were considered culturally incompatible and excluded. These four scenarios were number 11 from the compassionate category, number 1 from the honesty category, number 15 from the sneaky category, and number 5 from the unfaithful category. Then, the translations were back-translated to English by a native bilingual and it was checked by another translator, familiar with psychology, to see whether it was matched to the original English version of moral vignettes. There were no major semantic and linguistic differences between the back-translated English version and the original English form.

**Step 2. Gathering the data:** We asked the participants to read the paper-and-pencil moral vignettes in a quiet room in a timeless setting. They then rated each of the 150 scenarios on 13 dimensions on a 7-point Likert scale.

**Step 3. Exploratory factor analysis (EFA):** We performed an exploratory factor analysis on 10 out of the 13 dimensions of the moral vignettes [frequency, personal familiarity, and general familiarity were excluded since they were not considered as moral factors based on Knutson et al. (2010)]. The extraction method was based on eigenvalues > 1 and we used principal axis factoring. We used KMO and Bartlett's test for sphericity for sampling adequacy and the Promax rotation since the factors were not completely orthogonal (Russell, 2002). The Kaiser-Meyer-Olkin measure of sampling adequacy was 0.786 which is > 0.7 and shows that the sample size is adequate (Kaiser, 1974; Rajalahti and Kvalheim, 2011). We used SPSS version 26 to analyze the data.

**Step 4. Cross-study comparison of the underlying moral cognitive factors:** We then determined whether there are differences between the factors that were extracted comparing the EFA results in the current study with those of Knutson et al. (2010).

**Step 5. Shortening the questionnaire within each category:** Knutson's et al. questionnaire consists of 150 moral scenarios that measured 13 dimensions for each scenario. Thus, completing the questionnaire is time-consuming. Therefore, we decided to reduce the scenarios within each category. To that end, we separately conducted a factor analysis for each category and selected the scenarios in a way that conserved all of the factors. The number of factors differed among categories but the maximum number of factors in a category was four. So, we conducted the same factor analysis procedure in step 2 for each category (ten-factor analyses in total) and we set the number of extracted factors to four for each new category since the maximum number of extracted factors among all categories was four. Finally, we selected one scenario from each factor with the highest loading.

**Step 6. Cross-study comparison of the reduced questionnaire:** We did the same comparison as in step 3, this time with the reduced questionnaire.

**Step 7. Exploratory factor analysis of the categories:** The questionnaire had 10 categories (compassionate, guilty, honest, hurtful to someone, lied, regretful, sneaky, tempted, took something, unfaithful). One may ask whether these categories have some underlying cognitive factors or not. To test this, we used exploratory factor analysis on the 10 categories. The procedure was the same as step 3, however since the factors were orthogonal with the criterion (factor correlation matrix off-diagonal elements were below 0.3), we used varimax rotation (Russell, 2002).

**Step 8. Cronbach's alpha measurement:** To check the internal consistency of the questions in the questionnaire, and questions within each category, we measured Cronbach's alpha for the 150 scenarios, for the scenarios within each category, and the shortened questionnaire.

## Results

### Factor Analysis

#### Dimension Reduction on 10 Dimensions Out of 13 Dimensions

**Figure 1:**
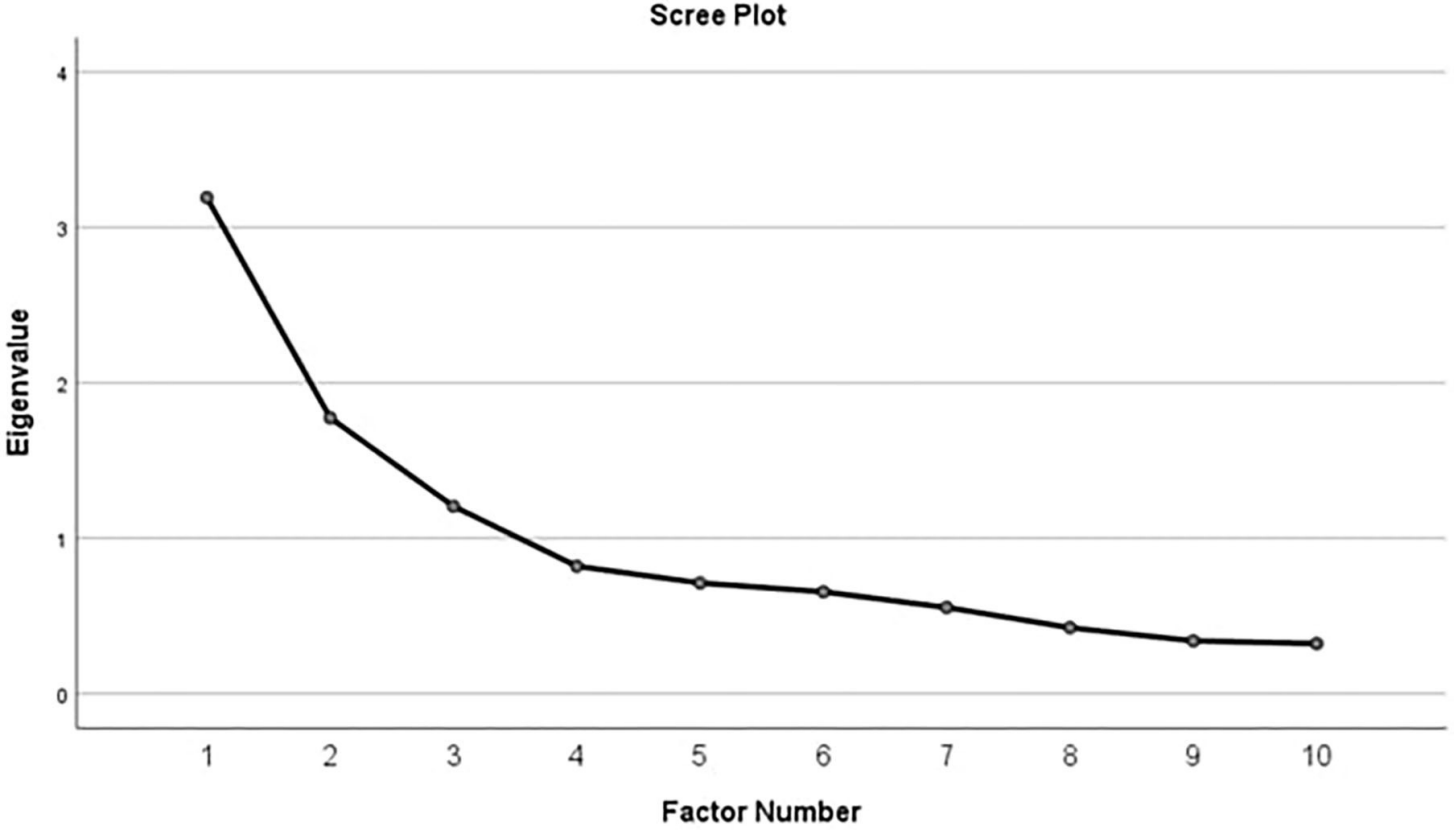
Determining the number of factors based on the scree plot.

We calculated subject ratings and conducted a factor analysis of the scenarios. Exploratory factor analysis (EFA) is a method of describing the observed variability among correlated dimensions and in our questionnaire through factors. For example, if the emotional intensity and emotional aversion dimensions correlate with each other in the participants' responses, it is possible that both of them are being derived by a mutual factor (e.g., social affect). In the current study, we sought to investigate the underlying cognitive moral factors for 10 dimensions out of the 13 total dimensions) identified by Knutson et al. (2010). Based on eigenvalues and the scree plot, 3 factors were identified (see Figure 1). Therefore, we continued with these 3 factors using the principal axis factoring method to form the pattern matrix (Russell, 2002).

Table 1 indicates the extracted factors based on the EFA method. We named these factors based on Knutson et al. (2010) who did a similar analysis. These three factors are norm violation, intention, and social affect. If we look at the loadings that each dimension has on the factors, we can see that social norm violation, moral appropriateness, illegality, harm to others, and other-benefit can be driven by the norm violation factor (all have loadings more than 0.4 on this factor).

**Table 1:** Pattern matrix for the three factors that were extracted through EFA method in Iran.

**Factors**	**Dimensions**	**Norm violation**	**Intention**	**Social affect**
Norm violation	Social norm violation	0.837	−0.011	−0.206
	Moral appropriateness	−0.814	−0.062	0.026
	Illegality	0.787	0.081	0.172
	Harm to others	0.539	0.125	0.471
	Other-benefit	−0.457	0.195	−0.017
Intention	Premeditation	−0.159	0.654	0.012
	Self-benefit	0.200	0.607	−0.055
Social affect	Emotional intensity	−0.159	−0.030	0.470
	Emotional aversion	0.367	−0.177	0.423
	**Socialness**	**−0.252**	**0.270**	**0.342**

*Bold values mean that none of the three underlying factors can drive subjects response to the socialness dimension*.

Subjects' response to the illegality dimension is driven by norm violation factor, and not by intention or social affect factors. Subjects perceived something as illegal if it violated the social norm.

The second factor, intention, was driven by the premeditation or self-benefit dimensions. Finally, emotional intensity and emotional aversion dimensions are being driven by the social affect factor.

#### Comparing the Factors Extracted in Iran With Factors Extracted in America

One of the most important questions in studying everyday moral decision making is whether culture can influence moral decisions. To that end, we investigated whether there is a difference between the underlying moral cognitive factors that were extracted in the current study with those factors that were extracted from the same questionnaire used in the Knutson et al. (2010) study. Table 2 represents these factors.

**Table 2:** Results of the factors analysis for the three factors that were extracted through EFA method in America (based on Knutson et al., 2010).

**Factors**	**Dimensions**	**Norm violation**	**Intention**	**Social affect**
Norm violation	Moral appropriateness	−0.956	−0.120	−0.102
	Social norm violation	0.947	0.144	0.154
	Other-benefit	−0.883	0.051	0.046
	Harm to others	0.803	0.009	0.473
	Illegality	0.737	0.115	−0.288
Intention	Premeditation	−0.002	0.859	0.175
	Self-benefit	0.244	0.772	−0.340
Social affect	Emotional intensity	0.024	−0.066	0.896
	Socialness	−0.115	0.154	0.763
	Emotional aversion	0.336	−0.258	0.762

Examining the exploratory factor analyses (EFA) in the two studies, it can be seen that the same three factors (norm violation, intention, and social affect) drive subjects' decisions. The “harm to others” dimension has a considerable loading on the social affect dimension, similar to the Iranian data. However, there are some notable differences. First, the order of the loadings on the norm violation factor is different in the two studies. Furthermore, the “socialness” dimension has a high loading on social affect in the American case whereas, in the Iranian data, this factor does not have a high loading on any of the dimensions.

#### Dimension Reduction in Each Category to Shorten the Questionnaire

The moral vignettes in the last two analyses, consisted of 150 moral scenarios (10 categories and 15 scenarios in each category), each examining 13 dimensions. If subjects are asked to make judgments about all the scenarios, the task would be very time-consuming. To shorten the task, we conducted exploratory factor analyses and reduced the number of questions from fifteen to four in each category (see the methods for a further explanation). Thus, instead of 150 scenarios, we were able to reduce the stimulus set to 40 scenarios. Table 3 shows the identification of the selected questions in each category.

**Table 3:** The reduced questionnaire based on exploratory factor analysis within each category.

**Category number and name**	**Scenario numbers**
Category 1, Compassionate	1, 8, 9, 15
Category 2, Guilty	18, 20, 23, 26
Category 3, Honest	33, 35, 37, 45
Category 4, Hurtful to someone	48, 50, 52, 59
Category 5, Lied	62, 66, 68, 69
Category 6, Regretful	76, 79, 82, 89
Category 7, Sneaky	92, 99, 101, 103
Category 8, Tempted	108,110,115,117
Category 9, Took something	125, 126, 128, 134
Category 10, Unfaithful	136, 141, 143, 148

#### EFA on the Shortened Questionnaire and Comparing It With the American Survey

Table 4 represents the underlying cognitive factors for the reduced questionnaire. The factors and the dimensions are similar to the ones extracted using the entire scenario set. However, the socialness factor which did not have a considerable loading on any of the factors in the previous analysis here helps drive the intention factor.

**Table 4:** The reduced questionnaire factors based on exploratory factor analysis.

**Factors**	**Dimensions**	**Norm violation**	**Intention**	**Social affect**
Norm violation	Social norms	0.866	0.120	0.065
	Legality	0.814	−0.091	0.186
	Moral judgment	0.782	−0.061	−0.064
	Harm to others	−0.430	0.232	0.411
	Other-benefit	0.426	0.156	−0.066
Intention	Self-benefit	−0.239	0.643	−0.122
	premeditation	0.112	0.618	−0.031
	**Socialness**	**0.302**	**0.459**	**0.185**
Social affect	Emotional aversion	−0.222	−0.172	0.620
	Emotional intensity	0.232	0.055	0.409

*Bold values mean that the socialness is being drived by intention factor in the reduced version of the questionnaire that is different from the results in Knutson et al*.

#### EFA in Categories

The last analysis we conducted determined whether the categories have the same underlying factors or not. To test this, we did an exploratory factor analysis on the categories. Since the scenarios were not comparable with each other, we used the mean of all 15 scenarios in each category to obtain a thirteen-dimension vector for each category. Then, we used the EFA method to find the underlying cognitive factors. Table 5 shows these two factors. The categories honest and compassionate have the same underlying factors, while another factor drives subjects' results in responding to the other 8 categories. By looking at the categories within each factor, we established that the two categories in factor number two are moral actions and the other eight categories in factor number one are immoral actions.

**Table 5:** The underlying cognitive factors of the categories based on an exploratory factor analysis.

**Factors**	**Categories**	**Immoral actions**	**Moral actions**
Immoral actions	Category 6, Regretful	0.852	0.053
	Category 8, Tempted	0.822	−0.145
	Category 4, Hurtful to someone	0.809	0.016
	Category 5, Lied	0.801	0.127
	Category 9, Took something	0.801	−0.414
	Category 7, Sneaky	0.785	−0.223
	Category 10, Unfaithful	0.753	−0.473
	Category 2, Guilty	0.674	0.008
Moral Actions	Category 3, Honest	0.088	0.840
	Category 1, Compassionate	−0.130	0.807

One might ask whether moral and immoral actions differ in terms of emotional arousal. We investigated this question by analyzing the difference in the mean of emotional intensity and emotional aversion. The results indicated that immoral actions had a higher emotional aversion in comparison with moral actions, but no difference was observed between emotional intensity in moral and immoral actions. The moral actions' mean emotional intensity was 4.06 (SD = 1.02) while mean emotional intensity for immoral actions was 3.68 (SD = 1.13) (two-sided paired *t-*test, *p* = *0.12, t-stat* = *1.6*). The mean emotional aversion for moral actions was 2.96 (SD = 0.41) while mean emotional aversion for immoral actions was 4.36 (SD = 0.72) (two-sided paired *t-*test, *p* = *0.0000000032, t-stat* = –*9.05*).

#### Cronbach's Alpha on 150 Scenarios

To use the moral scenarios in tasks, it is important to determine internal consistency across the scenarios (Cronbach, 1951; Reyes-Menendez et al., 2019). The Cronbach's alpha (Cronbach, 1951) measure for the whole 150 scenarios was 0.96. Also, we measured Cronbach's alpha for each category. These measures were 0.904, 0.826, 0.878, 0.897, 0.894, 0.877, 0.917, 0.893, 0.944, 0.966, for categories 1–10, respectively. All of the measures were above 0.7 which shows a good level of internal consistency (Nunnally and Bernstein, 1994) among all of the questions and the questions in each category. In addition, the Cronbach's alpha measure for the 40 scenarios in the reduced questionnaire was 0.895. Furthermore, the Cronbach's alpha for the categories in each scenario in the reduced version of the questionnaire were 0.533, 0.486, 0.602, 0.713, 0.619, 0.653, 0.728, 0.673, 0.818, and 0.866. Since the number of scenarios within each category was small, the low measures for Cronbach's alpha were expected.

## Discussion

The homo-economicus model that assumes human beings are self-interested creatures (Kargol-Wasiluk et al., 2018), one of the main cornerstones of mainstream economics, has been replaced by homo-moralis models (Kargol-Wasiluk et al., 2018). Moral judgment is a complex construct that involves different dimensions such as self and other-regarding motives, emotions, norms, etc. (Moll et al., 2005). To examine these various dimensions, many academic studies have exploited complex and rare situations that most people have not experienced and that require additional reasoning and other cognitive processes besides moral judgment. For many studies of moral judgment, it may be that using everyday moral dilemmas is more sensible (Knutson et al., 2010). No matter the moral scenario used, ecological, and cultural backgrounds may influence the interaction of the stimulus dimensions (Moll et al., 2005). In the current study, we investigated whether there is a difference between everyday moral judgments in Iranian culture, a non-western country, vs. those reported in American culture by Knutson et al. (2010). Comparing the two studies, the same dimensions, except the socialness dimension, loaded onto the same factors. This observation suggests that the moral judgments of everyday dilemmas in the two cultures were mostly similar.

Other regarding motives (harm to others and others' benefit), one of the moral dimensions in our study, was driven by social norms, and not by intentions which drives self-benefit motives. First, this observation was similar to the Knutson et al. (2010) findings. Second, this supports the view that subjects' self and other motives may be modulated by distinct brain areas and processes (Hutcherson et al., 2015). It might be interesting to compare the underlying factors in self and other-regarding motives in normal subjects vs. patients with self-and other related problems such as schizophrenia or borderline personality disorders (Bender and Skodol, 2007; Beeney et al., 2016; Fuentes-Claramonte et al., 2020).

Having illegality and moral appropriateness both drive the norm violation factor may imply an interplay of rules and morality in facing moral dilemmas. Emotion, as another dimension of a homo-moralis person (Kargol-Wasiluk et al., 2018), was one of the main preventers of harm to others but not for other-benefit judgments. Similar to the Knutson et al. (2010) study, harm to others had a considerable loading (0.471) on the social affect factor in the Iranian study. Thus, both social affect and norm violation influence subjects' behavior in “harm to others” situations. Although both other-benefit and harm to others are driven by the norm violation factor, the other-benefit dimension does not have a significant loading on the social affect factor. This emphasizes the role of norms play in societies to increase the overall benefit and asserts that people's cooperativeness can be reduced if norms do not support other benefit actions sufficiently (Rand and Nowak, 2013; Ackermann and Murphy, 2019). Furthermore, this observation indicated that other-regarding motives may be driven by two distinct moral dimensions: social affect (that drives harm to others' judgments) and social norm violation (that drives other-benefit as well as harm to others' judgments).

Regarding the investigation of underlying moral factors *differences* in the current study and those reported by Knutson et al. (2010), harm to others, other-benefit, emotional aversion, and emotional intensity had lower loadings in this study. One reason for this observation about harm to others and other benefit motives might be related to the role of religion in Iranian culture, i.e., the main causes for other-regarding behavior might not be social norms. Harm to others' aversion partly might originate from religious believes that altruistic choices come with a better position in the other life and God does not like wrongdoers. Although a considerable percentage of the American people are religious too (Ikenberry and Appiah, 2006; Bloom, 2012), it is not clear to what extent the American people consider religious beliefs in their moral actions compared to Iranians. Further experiments are needed to address this issue and compare the extent that Iranians and Americans consider religious tenets in their moral decisions and judgments. Furthermore, the relationship between morality and religion is not straightforward (Bloom, 2012; Norenzayan, 2014). To address these issues, one possible solution is to add religious violations as another dimension and administer the moral scenario survey to religious and non-religious people across cultures.

It is noteworthy to mention that the “socialness” dimension did not have a high loading (more than 0.4) on any of the three factors. One possible explanation for this result is that the meaning of the word “socialness” in Farsi language might not be clear; for example, it might have been perceived as someone being social, instead of involving social interactions. Therefore, subjects' might have not understood the desired meaning. In addition, the exploratory factor analysis resulted in two factors for the categories which are moral and immoral actions. We found that immoral actions have a higher emotional aversion in comparison with moral actions.

Finally, the data that has been collected in the current study does not come from a nationally representative sample in terms of age and ethnicity, or education. Further use of this survey with larger sample sizes from different ethnicities residing in Iran is needed to obtain a more precise view of Iranian beliefs about moral issues.

## Conclusions

Morality is a construct that includes various dimensions and whenever a person needs to make a moral judgment, s/he uses them. Our moral judgment task was not confined to rare dilemmas, rather, it consists of everyday social interactions. The majority of research in the domain of morality has used a limited number of moral dimensions and rare situations with limited cross-cultural reliability (Greene et al., 2001; Greene and Haidt, 2002; Graham et al., 2009, 2016). The current study aimed to resolve these contradictions by using everyday moral dilemmas with various dimensions and tried to investigate whether there were differences between the underlying moral cognitive processes between Iranian and American cultures (Knutson et al., 2010). Taken together, the results validated the role of three primary factors in moral decision making in Iran: norm violation, intention, and social affect. We showed that the same factors influence the same dimensions in Iranian and American cultures, with a minor difference in that socialness was not an underlying factor in our analysis. The validation of this questionnaire in Iran provides a tool for future researchers to study the moral judgments within everyday contexts. Furthermore abbreviating the long original version (almost 200 A4 pages) would decrease the risk of cognitive fatigue in answering moral scenarios which could impact moral judgment (Timmons and Byrne, 2019). It also makes it a good measure to study brain-injured patients who may have a reduced cognitive reservoir and are more vulnerable to cognitive fatigue (Kohl et al., 2009).

## Data Availability Statement

The original contributions presented in the study are publicly available. This data can be found here: https://figshare.com/search?q=10.6084%2Fm9.figshare.12768023.

## Ethics Statement

The studies involving human participants were reviewed and approved by the Institute for Research in Fundamental Sciences (IPM), Tehran, Iran. The participants provided their written informed consent to participate in this study.

## Open Practice Statement

Neither of the experiments reported in this article was formally preregistered. The data is available at doi: 10.6084/m9.figshare.12768023.

## Author Contributions

JG and FM developed the study concept. FM, KA, SSh, AJ, FG, PH, and PJ contributed to the study design and validation of the moral scenarios in Iranian culture. Testing and data collection were performed by SSo and PH. AY performed the data analysis and interpretation under the supervision of FM. AY and SSo drafted the manuscript. JG, FM, and SSh revised it. All authors contributed to the article and approved the submitted version.

## Conflict of Interest

The authors declare that the research was conducted in the absence of any commercial or financial relationships that could be construed as a potential conflict of interest.
